# Susceptibility to Electronic Cigarette and Consumption Patterns in Adolescents

**DOI:** 10.3390/nursrep14020098

**Published:** 2024-05-22

**Authors:** Esperanza Santano-Mogena, Sergio Rico-Martín, Cristina Franco-Antonio, Sergio Cordovilla-Guardia

**Affiliations:** 1 Nursing Department, Nursing and Occupational Therapy College, Universidad de Extremadura, Avda Universidad, s/n, 10003 Cáceres, Spainsergiorico@unex.es (S.R.-M.);; 2 Health and Care Research Group (GISyC), Universidad de Extremadura, 10003 Cáceres, Spain

**Keywords:** susceptibility, curiosity, extended index of susceptibility, vaping, electronic cigarette, adolescents

## Abstract

The use of electronic cigarettes (ECs) is a major problem globally. Susceptibility and curiosity are important factors that develop prior to the onset of substance use, such as ECs, and are therefore considered as predictors. Both factors are used to obtain an extended index of susceptibility (ESIe-c), which allows the identification of adolescents who are at risk of starting to use these devices. The aim of this study was to determine the prevalence of EC consumption and to assess the association of possible predictors with susceptibility to use and experimentation with ECs among adolescents. A cross-sectional study was carried out in 377 adolescents (53.0% female). Participants were compared according to their experimentation with ECs. A total of 45.9% had already used electronic cigarettes, and 20.8% were current users. Among those who had not tried electronic cigarettes, 43.3% had a medium-high susceptibility to EC initiation. Consumption among close friends, receiving offers of consumption and alcohol consumption were associated with initiation. There was an inverse association between a medium susceptibility index electronic cigarette score and the consumption of cigarettes and positive affect; on the other hand, the lack of premeditation was associated with a higher susceptibility index score. Intrapersonal factors and social factors seem to influence the ESIe-c and onset of EC use, respectively. The main conclusion in this study is that susceptibility is influenced by intrapersonal factors such as affectivity and impulsivity through the lack of premeditation, and by social factors such as EC consumption by best friends.

## 1. Introduction

Vaping, a term used to refer to the use of electronic cigarettes (ECs), has become a very widespread form of consumption among young people and adolescents in recent years, becoming an emerging problem in the field of public health [1,2]. EC use has substantially increased among nonsmoking adolescents [3]. According to data from the National Youth Tobacco Surveys (NYTSs), in 2021, ECs were the product most used by high school students [4]. In Spain, according to a national survey on the use of drugs in secondary education, 44.3% of students between 14 and 18 years of age had used ECs at some time, with 22.8% using ECs in the last year [5].

ECs emerged as a safe alternative to promote smoking cessation. EC use has negative health consequences including hemodynamic alterations and a negative impact on lung function [6,7]. Furthermore, in adolescents, exposure to nicotine can affect brain development and cognition [8].

The use of these devices seems to be influenced by sociodemographic, socioenvironmental and intrapersonal factors. The prevalence of consumption and frequency of use are higher in adolescents, an early initiation of EC use is associated with higher levels of consumption [9] and, regardless of the consumption pattern, males with higher socioeconomic status consume more [10].

Among socioenvironmental factors, consumption in the family environment and among friends and colleagues has been related to experimentation and subsequent use [11,12,13] and to two misperceptions, i.e., that EC use represents a lower risk to health and is more socially accepted than traditional cigarettes [12]. With respect to intrapersonal factors, a greater lack of premeditation and higher levels of negative urgency or the tendency to act under conditions of negative affect have been linked to vaping [14]. Greater impulsivity is associated with earlier onset and with a greater frequency of use [9]. Vaping has been associated with the presence of other risk behaviours, such as marijuana use [15,16].

Similarly to what occurs with cigarette smoking, before experimentation, susceptibility to smoking and a series of cognitions that increase the probability of smoking develop in adolescents [17,18,19]. Susceptibility to smoking is defined as the absence of a firm commitment not to smoke. This construct, developed by Pierce et al. [20], has been widely used, both in cross-sectional and longitudinal studies, in the identification of adolescents and young people who do not smoke but are at risk of initiating the consumption of cigarettes and other alternative products [18,19]. Another cognition element related to the consumption of tobacco products is curiosity. Based on these predictors of initiation, a new measure has been developed, an extended susceptibility index [21], which improves the identification of adolescents at risk of initiating use [17]. Several investigations have explored the predictive validity of this construct [17,19].

This predictor of initiation of consumption, the extended index of susceptibility, in the case of EC consumption, has been associated in previous research with not having previously tried nicotine, with consumption in the family or social environment and with other risky behaviours such as alcohol consumption [19,22].

Young people have greater susceptibility to ECs [22], which translates into a higher risk of initiating consumption [17,19]. There is evidence to suggest that young people who have never smoked and who have tried ECs are more likely to smoke cigarettes [23] and progress to smoking cigarettes consistently [1]. This would put at risk the achievements made in the fight against the tobacco epidemic.

In Spain, no studies have been found that assess susceptibility to the use of ECs or that explore the variables related to this initial predictor. It has been suggested that these factors could be specific to each country or region [24]. Therefore, it is relevant to explore both the pattern of EC consumption in adolescents and the variables that are associated with high susceptibility to the initiation of consumption.

The aims of this study were to determine the patterns of EC consumption and to analyze the susceptibility to ECs in nonsmoking adolescents in order to assess the association of possible predictors with the susceptibility and experimentation with ECs among adolescent aged 12–16 years.

## 2. Methods

### 2.1. Design

A cross-sectional study was carried out from November 2019 to February 2020 in which 2nd–4th grade compulsory secondary education students from various educational centers in a city located in western Spain participated.

### 2.2. Participants and Data Collection

From a list of centers, three educational centers were randomly selected through computer software. The centers were contacted and, after authorization was obtained from the board of directors, all students enrolled in the 2nd (13–14 years), 3rd (24–15 year) and 4th (15–16 years) of compulsory secondary education were offered the possibility of participating in the study. Informed consent was requested through letters to the parents, guardians or legal representatives. Parental and minor consent were obtained before the study began. Those students who provided parental consent were invited to complete voluntarily a computer-assisted self-administered questionnaire. During this process, the participants could ask for assistance from a researcher to resolve questions.

### 2.3. Ethical Considerations

The internal review board of the Extremadura University approved the research protocol (Cod. 187/2019). All the participants were informed about the objectives of the study and the research methods applied; they were asked to provide informed consent forms signed by their parents. The participation of the students was voluntary, and they were informed that they could refuse to participate and leave the study at any time.

### 2.4. Validity and Reliability

#### 2.4.1. Main Variables in the Study

Susceptibility to vaping was evaluated according to the algorithm developed by Pierce et al., adapted to ECs, that assesses future intention, intention to use and social influence [20] by asking the following questions: (i) “Do you think you will experiment with EC in the future?”, (ii) “At any time during the next year, do you think you will smoke an EC?” and (iii) “If one of your best friends offered you an EC, would you smoke it?”. The range of responses was “Definitely no”, “Probably no”, “Probably yes” and “Definitely yes”. Those participants who answered “Definitely no” to all the questions were classified as “not susceptible”, and those who answered “Probably yes” or “Definitely yes” to any of the three questions were classified as highly susceptible. The rest were considered “susceptible” [20].

To assess curiosity, the following question was used: “Have you ever been curious about EC?” A range of responses was established from “Definitely no”, “Probably no”, “Probably yes” and “Definitely yes”. Those participants who answered “Definitely no” were classified as not curious, those who answered “Most likely, not” were classified as curious, and those who answered “Probably yes” or “Definitely yes” were classified as highly curious [21].

From susceptibility and curiosity, the expanded susceptibility index electronic cigarettes (ESIe-c) was obtained. The participants who answered “Definitely no” to the questions of susceptibility and curiosity were classified as “Low susceptibility” or “Not susceptible”, those who answered “Probably yes” or “Definitely yes” to some of the questions were classified as “Highly susceptible”, and the remaining individuals were classified as “Susceptible”. This index has an internal reliability of 0.74 in adolescents aged from 12 to 17 years [21].

Experimentation with ECs was determined using the following questions: “Have you ever tried or experimented with e-cigarette smoking, even a few puffs?” [25]; if the response was “yes”, a second question was asked: “Have you ever smoked EC? Do not answer ‘yes’ if you only took a few puffs of someone else’s EC”. To identify current smokers, a question was asked about EC use in the previous 30 days [26]. Participants were also asked about the age at which the experimentation occurred and the start of use.

#### 2.4.2. Independent Variables

Participants reported sociodemographic data, including age, gender (male, female, other), composition of the family unit and the educational level of their parents, which vas determined using a scale with different options ranging from “Never studied to University studies”. The socioeconomic status of the family unit was assessed with the family affluence scale (FASII), which assesses family wealth. The FAS II scale consists of four items; each item had several response options that was assigned a score. The scores were summed and family affluence was classified as low when the score was 0–2 points, average when the score was 3–5 points and high when the score was 6–9 points [27].

The participants were asked about the state of consumption of conventional cigarettes and hookah and about their perception of risk related to the possibility of developing a health problem derived from the use and consumption of ECs. Included the following response options: “It will not happen”, “Not likely”, “Likely”, “Very likely”, “It will definitely happen” and “I do not know” [28]. Other behaviours, such as alcohol and cannabis consumption in the previous 30 days, were explored using timeline follow-back (TLFB). The AUDIT-C scale was applied to identify risky alcohol consumption. This scale has an internal consistency (Cronbach’s alpha) of 0.82 in the adolescent population [29].

In the social and family environment, the perception of electronic and conventional cigarette consumption by members of the family unit, the person’s five best friends and their classmates was assessed. The response was classified into five categories (“Hardly anyone, Less than half, half, More than half and Almost everyone”). Exposure to secondhand smoke in the home was assessed in the week prior to the study, and the participants were asked about the frequency of consumption offers in the previous 30 days [30].

The intrapersonal variables impulsivity and affectivity were considered. Impulsivity was assessed with the impulsive behaviour scale (UPPS-P), which evaluates four dimensions of this variable: urgency, sensation seeking, lack of perseverance and lack of premeditation. In the adolescent population, the internal consistency (Cronbach’s alpha) was greater than 0.7 in adolescents aged from 11 to 17 years [31]. Affectivity was evaluated with the Affectivity Scale (PANAS), which assesses positive and negative affect. The internal consistency of this scale in the adolescent population was 0.72–0.73 for positive affect and 0.74–0.75 for negative affect in girls and boys with an age range from 12 to 17 years, respectively [32].

#### 2.4.3. Statistical Analyses

Descriptive analyses allowed us to verify the distribution of the variables in the entire sample. As measures of central tendency, for the quantitative variables, the mean and standard deviation (±SD) were used when the data presented a normal distribution, and the median and interquartile range [IQR] were used when the data presented a nonnormal distribution. To compare the quantitative variables, data with a normal distribution were compared using Student’s *t* test, and data with a nonnormal distribution were compared with the Mann-Whitney U test. Pearson’s chi-square test was used to compare the categorical variables. A multinomial logistic regression model was used to examine the association between ESIe-c, categorized into three levels (not susceptible, susceptible and highly susceptible), and the independent variables. From these analyses, the cOR (crude odds ratio) and aOR (adjusted odds ratio) and their respective 95% confidence intervals were obtained.

## 3. Results

A total of 488 students from the 2nd to 4th grades of compulsory secondary education were invited to participate; parental consent was obtained for 436 (89.3%) students. With respect to the excluded participants, 19 (4.4%) declined to participate and 40 (8.1%) participants presented missing data and outlying data. The final analytical sample consisted of 377 (77.0%) students. The median age of the participants was 15 [14–15] years; of those who reported their gender, 53.0% were female.

Regarding consumption patterns, 45.9% (*n* = 173) indicated some level of experimentation or consumption. Of those who had experimented, 20.8% (*n* = 36) were current users, and 204 (54.1%) had not tried ECs.

Regarding the sociodemographic variables (Table 1), the adolescents who had experimented with ECs were older (15 [14–15] years old) than those who had not tried ECs (14 [13–15] years old) (*p* < 0.001). Those who had experimented were mostly female (98 (58.8%) females and 68 (41.2%) males, *p* = 0.045). Differences were found in the composition of the family unit: those who had experimented reported living at home with multiple people rather than few people (55 (31.8%) versus 42 (20.6%), *p* = 0.013), and had a lower family purchasing power than did those who had not tried ECs.

When exploring the use of other products and substances (Table 1), higher consumption was observed for all the products explored among the adolescents who had experimented. For conventional cigarettes and hookah, 61 (35.3%) and 42 (24.3%) of those who had experimented with ECs had consumed these products, respectively, compared to 16 (7.8%) and 14 (6.9%) of those who had not tried ECs (*p* < 0.001). In addition, those classified as experimenters reported higher cannabis use (26 (15.0%) compared to 4 (2.0%), *p* < 0.001), and alcohol consumption than did those who had not tried ECs, with 46 (26.6%) of those who experimented presenting risky alcohol consumption, as assessed with the AUDIT-C scale, and 7 (3.4%) of those who had not tried ECs presenting risky alcohol consumption (*p* < 0.001).

Regarding intrapersonal variables (Table 1), adolescents who had tried ECs scored higher in the dimensions of urgency, lack of perseverance and global impulsivity than did those who had not tried ECs, with medians of 24 [21–27] vs. 22 [18–25] (*p* < 0.001), 8 [6–10] vs. 8 [6–9] (*p* = 0.004) and 56 [50–63] vs. 51.5 [44–59] points (*p* < 0.001), respectively. With respect to affectivity, adolescents who were EC users had higher scores for negative affect and total affectivity than did those who had not tried ECs (21 [18–23] points vs. 20 [17–22], *p* = 0.002 and 45 [42–48] points vs. 44 [41–46], *p* = 0.019, respectively).

From the exploration of the family and social environment (Table 2), differences were observed in the consumption of ECs based on family environment, with respect to siblings and other partners: 5.2% (*n* = 9) of those who had experimented reported consumption by siblings and among other partners, and 2% (*n* = 1) of students who had not tried ECs reported EC use by siblings and partners (*p* = 0.0027). A total of 13.3% (*n* = 23) of those who had experimented lived in the home with a user of ECs, while 3.4% (*n* = 7) of those who had not tried ECs lived in the home with a user of ECs (*p* < 0.001). With respect to the exposure to secondhand smoke in the home, the students who had experimented reported higher percentages of exposure for all the time frames included, and 58.5% (*n* = 101) of current EC users had not been exposed to secondhand smoke, compared with 74% (*n* = 151) of the participants who had not tried ECs (*p* = 0.005). Differences were also observed in the perception of consumption at home: 83.3% of the students who had not tried ECs reported that almost no one in their family environment used ECs, compared to 67.6% of those who had experimented with ECs (*p* = 0.004). Regarding the social environment, consumption within the group of the five best friends was higher in the group of those who had used ECs: 41.6% of those who had experimented had one or more friends who vaped, compared to 18.1% of those who had not tried ECs (*p* < 0.001). Regarding consumption offers, significant differences were found: 22.0% (*n* = 38) of those who had experimented had received offers in the previous 30 days, compared to 4.2% (*n* = 10) of students who had not tried ECs (*p* < 0.001). With regard to the perceived risk derived from consumption, no differences were found between the two groups.

**Figure 1:**
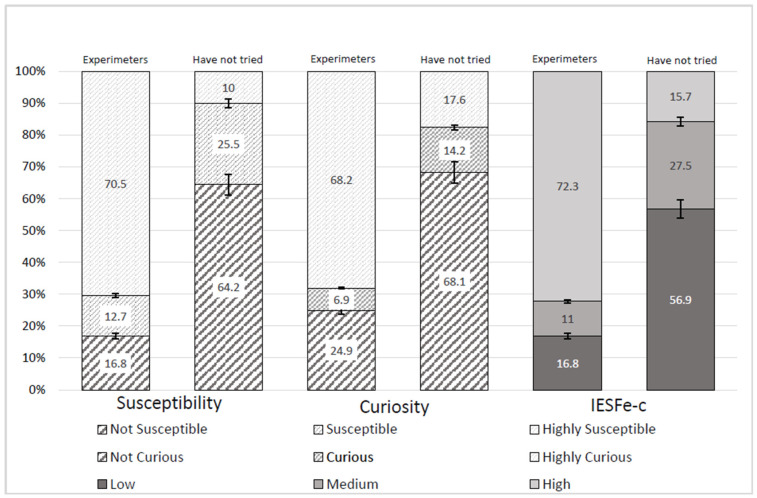
Susceptibility, curiosity and ESIe-c. Note: The first column for each variable (susceptibility, curiosity and ESIe-c) corresponds to the participants who had experimented with vaping, and the second column corresponds to those who had not tried electronic cigarettes.

From the analysis of susceptibility, curiosity and ESIe-c (Figure 1), higher levels of susceptibility to and curiosity about ECs were found among those who had experimented: in this group, 70.5% and 68.2% were categorized as highly susceptible and highly curious, respectively, compared with 10.0% and 17.6%, respectively, of those who did not use ECs (*p* < 0.001). The combination of both predictors in the ESIe-c yielded significant differences, with a high level of susceptibility (72.3%) among those who had experimented and a lower level (15.7%) among those who had not experimented (*p* < 0.001).

The results of the first multivariate analysis model of the association between experi-mentation and the different variables (Table 3) showed positive associations between ex-perimentation and age, consumption in a family and social environment, consumption of other products, alcohol and cannabis, and consumption offers. The variables with the greatest strengths of association were cannabis use and alcohol consumption (cOR: 8.84 [95% CI: 3.02–25.88], *p* < 0.001; cOR: 7.85 [95% CI: 4.93–12.49], *p* < 0.001 and cOR: 6.40 [95% CI: 2.84–8.29], *p* < 0.001 for cannabis use, alcohol consumption and experimentation, respectively). Among the sociodemographic variables, being of male gender was inversely associated with experimentation, with a cOR of 0.96 [95% CI: 0.43–0.99] (*p* = 0.045). Among the intrapersonal variables, the dimensions urgency, lack of perseverance and lack of premeditation were positively associated with experimentation, with values of cOR: 1.11 [95% CI: 1.06–1.59] (*p* < 0.001), cOR: 1.11 [95% CI: 1.03–1.12] (*p* = 0.007) and cOR: 1.07 [95% CI: 1.04–1.14] (*p* = 0.046), respectively. Regarding affectivity, only negative affect was positively associated with experimentation, with a cOR value of 1.102 [1.04–1.17] (*p* = 0.001). The adjusted analyses showed a strong association between alcohol consumption and experimentation (aOR: 4.249 [95% CI: 2.273–7.949], *p* < 0.001), followed by consumption and consumption offers by the five best friends (aOR: 3.778 [95% CI: 1.502–9.50] 4, *p* = 0.005; and aOR: 2.735 [95% CI: 1.479–5.06], *p* = 0.001, respectively).

The second multivariate analysis model analyzed the association between the susceptible and highly susceptible categories of the ESIe-c and the different independent variables (Table 4). The raw analyses showed that the urgency dimension was positively associated with susceptibility, with a cOR value of 1.07 [95% CI: 1.01–1.15] (*p* = 0.032), and that alcohol consumption was strongly associated with being highly susceptible (cOR: 3.73 [95% CI: 1.57–8.86], *p* = 0.003) and the dimensions of urgency, lack of perseverance and lack of premeditation. The adjusted analyses showed that cigarette consumption was inversely associated with susceptibility (aOR: 0.09 [95% CI: 0.008–0.98], *p* = 0.048) and the lack of premeditation was associated with high susceptibility (aOR value of 1.31 [1.09–1.59], *p* = 0.005).

## 4. Discussion

The purpose of this study was to determine the prevalence of EC use and examine the sociodemographic, socioenvironmental and intrapersonal characteristics of adolescents based on their levels of susceptibility and experimentation with this product. We found that 45.88% of the participants had initiated EC consumption, and among those who had not experimented with ECs, 43.3% presented a medium-high level of susceptibility according to the ESIe-c. Multivariate analyses showed an association between different intrapersonal, environmental and consumption variables with an increasing level of susceptibility and experimentation. The findings are consistent with the results of other investigations conducted on the susceptibility to vaping and the initiation of vaping in the adolescent population but also differ in some respects.

Regarding susceptibility, in this study, there was an inverse association between the consumption of conventional tobacco and a medium level of susceptibility but not with a high level of susceptibility. Polydrug use of tobacco products, cigarettes and other alternative products by the young population is widely documented [33]. Cigarette users seem to tend to consume other combustible products rather than noncombustible products such as ECs. Thus, in other studies, it has been found that young smokers with a low level of cigarette consumption had a lower susceptibility to vaping [34] and lower expected use of these devices [33].

Regarding the intrapersonal variables associated with the susceptibility index, we found that a higher positive affect implied a lower susceptibility to vaping (aOR: 0.83 [95% CI: 0.73–0.96], *p* = 0.010) and that a higher score for lack of premeditation was positively associated with being highly susceptible (aOR: 1.31 [1.09–1.5], *p* = 0.005), with a higher risk of initiating the use of these devices. These results are consistent with the results of other investigations that suggest that high scores for negative affect and low scores for positive affect are related to a greater risk of initiating the consumption of tobacco products, in addition to lower positive affectivity and a greater risk of consuming one or more tobacco products [35]. The association between a greater lack of premeditation, being more impulsive and high levels of susceptibility is in line with the results of recent investigations that show that the presence of internalization problems is related to a greater susceptibility to consumption in the adolescent population [36]. Thus, the consumption of these products could become a coping strategy for adolescents.

With regard to the initiation of consumption, the results obtained show that those who had experimented were mostly older girls, adolescents living with other EC users and those with greater exposure to secondhand smoke. Previous studies have reported an older age for EC users than for those who have not tried ECs [37,38]. However, these studies did not find greater consumption in females. In this regard, in our environment, the results of different national surveys carried out among young people and adolescents reflect a higher consumption among women, and research carried out in other regions suggests a similar proportion of initiators [37] or higher consumption by males [10,39].

Although consumption in the family environment and within the social environment favors initiation [13,28], the results obtained show that only vaping within the group of the five best friends and receiving consumption offers were associated with experimentation. These results are in line with other research findings that suggest that having a social environment where the consumption of electronic cigarettes is supported has positive effects both on initial use [40] and on subsequent consumption [41]. This effect could be linked to the existence of consumption offers within this social group because friends have been identified as one of the priority ways to obtain cigarettes, as have members of the family unit [39]. Adolescents, due to their age, cannot access these products at conventional points of sale; therefore, the offers they receive within their group of friends provide adolescents with an access route to the product [30] increasing the chances that the adolescent will start vaping.

Finally, in our study, we observed that those who had experimented had other risk behaviours, such as alcohol consumption. The association between the consumption of ECs and the presence of other risk behaviours, such as the consumption of other tobacco products, alcohol and other substances, in adolescents has been widely reported. Two studies conducted in the adolescent population reported that those who consumed alcohol had greater access to and probability of consuming ECs [42,43]. Among young people, alcohol consumption usually occurs within the social environment, among friends, and in these situations, it is likely that adolescents will receive offers to consume ECs, thus increasing the probability that they will start vaping.

Adolescence is a key period in human development, characterized by greater emotional reactivity along with increased social interactions [44]. All this could lead an adolescent who is immersed in a situation of emotional excitement and under the influence of a social environment that supports vaping to assume a risk such as experimenting or taking the first puffs on an EC.

The results of this study have relevance to clinical nursing practice. Nurses play an important role in health education and the prevention of toxic habits. The findings of this study have detected some factors that are associated with the likelihood of initiation of EC use, so nurses should emphasize these factors in order to prevent initiation.

## 5. Limitations

This study was carried out with students in the 2nd–4th grades of compulsory secondary education who resided in a region of western Spain. The selection of the participating schools was not carried out randomly, which could affect the external validity of the results. Regarding the design, it was a cross-sectional study that identified the existence of associations between the variables studied; however, causality could not be determined. The data were collected through self-reports, an approach that could affect the robustness of the results; however, in educational centers, this approach has been considered adequate to evaluate consumer behaviours [45]. In addition, anonymity and confidentiality supported the validity of the data collected.

## 6. Conclusions

The findings of this study suggest that both the susceptibility to vaping and the initiation of consumption were influenced by different factors. Susceptibility was influenced by intrapersonal factors such as affectivity and impulsivity through the lack of premeditation. Furthermore, social factors such as consumption among the individual’s five best friends and receiving offers of consumption influenced the initiation of EC use, along with other risk behaviours such as alcohol consumption. These findings suggest the need to design preventive programs taking into account the factors influencing this process; thus, it would be of significance for these programs to include components of affect regulation and impulsivity and component to reduce the influence of the social environment. The results of this study provide a guide for future longitudinal studies to confirm the associations detected, thus promoting more effective interventions.

## Figures and Tables

**Table 1:** Distribution of the sociodemographic and consumption variables in the entire sample and according to experimentation with electronic cigarettes.

	Total (*n* = 377)	Experimenters (*n* = 173)	Have Not Tried (*n* = 204)	*p*
**Age (years)** median [IQR]	15 [14–15]	15 [14–15]	14 [13–15]	**<0.001**
**Sex** (*n* = 364) *n* (%)				
Male	171 (47.0)	68 (41.2)	103 (51.8)	**0.045**
Female	193 (53.0)	97 (58.8)	96 (48.2)	
**Family composition**				
Mother	362 (96.1)	165 (95.4)	197 (96.6)	0.555
Father	328 (87.0)	148 (85.5)	180 (88.2)	0.440
Siblings	301 (79.6)	138 (79.8)	163 (79.9)	0.974
Grandparents	147 (39.0)	73 (42.2)	74 (36.3)	0.240
Other cohabitants	97 (25.7)	55 (31.8)	42 (20.6)	**0.013**
**Mother’s education level** (*n* = 362) *n* (%)				
University studies	152 (42.0)	60 (36.4)	92 (46.7)	
Secondary studies	127 (35.1)	58 (35.2)	69 (35.0)	0.127
Primary studies/no studies	83 (22.9)	47 (28.5)	36 (18.3)	
**Father’s educational level** (*n* = 328) *n* (%)				
University studies	118 (36.0)	46 (31.3)	72 (40.0)	0.297
Secondary studies	114 (34.8)	52 (31.5)	62 (34.4)	
Primary studies/no studies	96 (29.3)	50 (33.4)	46 (25.6)	
**Family affluence** *n* (%)				
Low	11 (2.9)	2 (1.2)	9 (4.4)	**0.044** ^1^
Medium	133 (35.3)	70 (40.5)	63 (30.9)	
High	233 (61.8)	101 (58.4)	132 (64.7)	
**Consumption in the last 30 days** *n* (%)				
Cigarettes	77 (20.4)	61 (35.3)	16 (7.8)	**<0.001**
Hookah	56 (14.9)	42 (24.3)	14 (6.9)	**<0.001**
Cannabis	30 (7.9)	26 (15.0)	4 (2.0)	**<0.001**
**Frequency of consumption of alcoholic beverages** *n* (%)				
Never	219 (58.1)	57 (32.9)	162 (79.4)	**<0.001** ^1^
One or fewer times a month	96 (25.5)	63 (36.4)	33 (16.2)	
2–4 times a month	53 (14.1)	44 (25.4)	9 (4.4)	
2–3 times a week or more	9 (2.4)	9 (5.2)	0 (0.0)	
**AUDIT-C test**	53 (14.1)	46 (26.6)	7 (3.4)	**<0.001**
**UPPS-P Impulsive Behaviours Scale** median [IQR]				
Urgency	23 [19–16]	24 [21–27]	22 [18–25]	**<0.001** ^2^
Sensation seeking	13 [10–16]	14 [10–17]	13 [9–16]	0.094 ^2^
Lack of perseverance	8 [6–10]	8 [6–10]	8 [6–9]	**0.004** ^2^
Lack of premeditation	10 [8–12]	10 [8–13]	10 [8–12]	0.069 ^2^
Total	54 [47–60]	56 [50–63]	51.5 [44–59]	**<0.001** ^2^
**Affectivity Scale (PANAS)** Median [IQR]				
Positive	24 [22–27]	23 [22–26]	25 [23–27]	0.282 ^2^
Negative	20 [17.5–22.5]	21 [18–23]	20 [17–22]	**0.002** ^2^
Total	44 [42–47]	45 [42–48]	44 [41–46]	**0.019** ^2^

Note: IQR: Interquartile range; AUDIT-C: Alcohol Use Disorder Identification Scale; UPPS-P: Impulsive Behaviours Scale; PANAS: Positive and Negative Affective Scale. ^1^ Fisher’s exact test, ^2^ Mann–Whitney U test. Significant results appear in bold.

**Table 2:** Consumption of electronic cigarettes and perceived risk of consumption in family and social settings.

	Total (*n* = 377)	Experimenters (*n* = 173)	Have Not Tried (*n* = 204)	*p*
**EC consumption in the family environment** *n* (%)				
Mother (*n* = 362)	1 (0.3)	1 (0.6)	0 (0.0)	0.455 ^1^
Father (*n* = 328)	5 (1.5)	3 (1.7)	2 (1.0)	0.664 ^1^
Siblings (*n* = 301)	11 (3.6)	9 (5.2)	2 (1.0)	**0.027** ^1^
Grandparents (*n* = 147)	2 (1.4)	1 (0.6)	1 (0.5)	1.000 ^1^
Other cohabitants (*n* = 97)	11 (11.3)	9 (5.2)	2 (1.0)	**0.027** ^1^
**Exposure to secondhand smoke in the home (last 7 days)** *n* (%)				
None	252 (66.8)	101 (58.4)	151 (74.0)	
1–2 days	31 (8.2)	16 (9.2)	15 (7.4)	
3–4 days	18 (4.8)	13 (7.5)	5 (2.5)	**0.005** ^1^
5–6 days	10 (2.7)	8 (4.6)	2 (1.0)	
7 days	66 (17.5)	35 (20.2)	31 (15.2)	
**Perception of EC consumption in the family environment** *n* (%)				
Hardly anyone	287 (76.1)	117 (67.6)	170 (83.3)	
Less than half	45 (11.9)	25 (14.4)	20 (9.8)	
Half	21 (5.6)	13 (7.5)	8 (3.9)	**0.004** ^1^
More than half	10 (2.7)	7 (4.0)	3 (1.5)	
Almost everyone	14 (3.7)	11 (6.4)	3 (1.5)	
**Perception of EC consumption among classmates** *n* (%)				
Hardly anyone	266 (70.6)	112 (64.7)	154 (75.5)	
Less than half	74 (19.6)	39 (22.5)	35 (17.2)	
Half	23 (6.1)	14 (8.1)	9 (4.4)	0.083 ^1^
More than half	10 (2.7)	7 (4.0)	3 (1.5)	
Almost everyone	4 (1.1)	1 (0.6)	3 (1.5)	
**Consumption by 5 best friends** *n* (%)				
None	268 (71.1)	101 (58.4)	167 (81.9)	
1–2	71 (18.8)	41 (23.7)	30 (14.7)	**<0.001** ^1^
3–4	22 (5.8)	17 (9.8)	5 (2.5)	
All	16 (4.2)	14 (8.1)	2 (1.0)	
**Risk perception** *n* (%)				
Will not happen	40 (10.6)	18 (10.4)	22 (10.8)	
Not likely	64 (17.0)	37 (21.4)	27 (13.2)	
Likely	119 (31.6)	51 (29.5)	68 (33.3)	0.396
Very likely	70 (18.6)	28 (16.2)	42 (20.6)	
It will definitely happen	16 (4.25)	7 (4.0)	9 (4.4)	
I don’t know	68 (18.0)	32 (18.5)	36 (17.6)	

^1^ Fisher’s exact test. Significant results appear in bold.

**Table 3:** Multivariate analysis 1. * Factors associated with experimentation with electronic cigarettes in the entire sample.

Exposure	cOR	(95% CI)	*p*	aOR *	(95% CI)	*p*
**Gender**						
Female	1.00 Ref			1.00 Ref		
Male	**0.65**	**0.43–0.99**	**0.045**	0.93	0.53–1.63	0.798
**Age** (1-year increase)	**1.475**	**1.23–1.77**	**<0.001**	1.10	0.79–1.31	0.890
**FAS** II						
Low–medium	1.00 Ref			1.00 Ref		
High	0.76	0.504–1.16	0.208	0.65	0.38–1.12	0.121
**Social factors** (increase in 1 smoker in the family or social environment)						
Family members	**4.10**	**1.71–9.85**	**0.002**	2.486	0.78–7.93	0.124
Close friends	**3.22**	**2.02–5.13**	**<0.001**	**2.735**	**1.48–5.06**	**0.001**
Classmates	1.836	0.92–3.66	0.085	0.895	0.36–2.21	0.809
**Smoke**						
Cigarette						
No	1.00 Ref			1.00 Ref		
Yes	**6.40**	**3.52–11.64**	**<0.001**	2.138	0.95–4.80	0.065
Hookah						
No	1.00 Ref			1.00 Ref		
Yes	**4.35**	**2.84–8.29**	**<0.001**	1.841	80.76–4.443	0.175
**Consumption of alcohol**						
No	1.00 Ref			1.00 Ref		
Yes	**7.85**	**4.93–12.49**	**<0.001**	**4.249**	**2.27–7.95**	**<0.001**
**Consumption of cannabis**						
No	1.00 Ref			1.00 Ref		
Yes	**8.84**	**3.02–25.88**	**<0.001**	3.782	0.89–16.5	0.071
**Electronic cigarette consumption offers**						
No	1.00 Ref			1.00 Ref		
Yes	**5.46**	**2.63–11.34**	**<0.001**	**3.778**	**1.50–9.50**	**0.005**
**Risk perception**						
Will not happen/not likely	1.00 Ref			1.00 Ref		
Likely to happen/definitely will happen	0.76	0.47–1.06	0.094	0.953	0.56–1.63	0.953
**UPPS-P (1-point increase)**						
Urgency	**1.11**	**1.06–1.59**	**<0.001**	1.008	0.95–1.07	0.805
Sensation seeking	1.042	0.99–1.09	0.085	1.042	0.98–1.11	0.196
Lack of perseverance	**1.11**	**1.03–1.12**	**0.007**	1.072	0.96–1.02	0.238
Lack of premeditation	**1.07**	**1.001–1.14**	**0.046**	0.957	0.87–1.06	0.379
**PANAS (1-point increase)**						
Positive affect	0.97	0.91–1.03	0.367	1.017	0.92–1.12	0.735
Negative affect	**1.102**	**1.04–1.17**	**0.001**	1.074	0.99–1.17	0.100

*n* = 377; * Binary logistic regression with experimentation as the dependent variable (category has not been tested as a reference) and the rest of the variables as independent variable. aOR: adjusted odds ratio. cOR: Crude odds ratio. 95% CI: 95% confidence interval. Ref.: Reference. UPPS-P: Impulsive Behaviours Scale. PANAS: Positive and Negative Affective Scale.

**Table 4:** Multivariate analysis 2. * Factors associated with ESIe-c in students who have not tried electronic cigarettes.

	Expanded Susceptibility Index e-c							
	Susceptible				Highly Susceptible			
	cOR (95% CI)	*p*	aOR * (95% CI)	*p*	cOR (95% CI)	*p*	aOR * (95% CI)	*p*
**Gender**								
Female								
Male	0.79 (0.39–1.41)	0.356	0.77 (0.37–1.60)	0.480	0.61 (0.27–1.37)	0.228	0.81 (0.29–2.32)	0.701
**Age (1-year increase)**	1.22 (0.86–1.47)	0.402	1.13 (0.82–1.56)	0.445	1.09 (0.79–1.52)	0.593	0.89 (0.55–1.44)	0.624
**FAS II**								
Low–medium	1.00 Ref		1.00 Ref		1.00 Ref		1.00 Ref	
High	0.78 (0.40–1.52)	0.467	0.80 (0.38–1.69)	0.564	0.60 (0.27–1.34)	0.214	0.55 (0.20–1.51)	0.246
**Social factors** (increase in 1 smoker in the family or social environment)								
Family members	3.23 (0.52–19.88)	0.207	3.73 (0.51–27.18)	0.194	3.80 (0.51–28.10)	0.191	3.86 (0.41–36.05)	0.236
Close friends	1.39 (0.62–3.12)	0.421	1.47 (0.56–3.87)	0.438	1.18 (0.43–3.25)	0.752	1.69 (0.45–6.31)	0.435
Classmates	2.20 (0.38–7.16)	0.190	2.07 (0.53–8.09)	0.296	1.90 (0.45–8.05)	0.385	2.07 (0.35–12.14)	0.419
**Consumption of alcohol**								
No	1.00 Ref		1.00 Ref		1.00 Ref		1.00 Ref	
Yes	1.33 (0.58–3.05)	0.499	1.63 (0.54–4.97)	0.388	**3.73 (1.57–8.86)**	**0.003**	3.09 (0.77–12.44)	0.112
**EC consumption offers**								
No	1.00 Ref		1.00 Ref		1.00 Ref		1.00 Ref	
Yes,	1.58 (0.34–7.34)	0.556	1.51 (0.23–10.19)	0.670	2.90 (0.61–13.67)	0.179	4.44 (0.57–34.18)	0.156
**Smoke**								
Cigarette								
No	1.00 Ref		1.00 Ref		1.00 Ref		1.00 Ref	
Yes	0.17 (0.22–1.38)	0.098	**0.09 (0.008–0.98)**	**0.048**	1.36 (0.40–4.61)	0.618	0.58 (0.10–3.41)	0.550
Hookah								
No	1.00 Ref		1.00 Ref		1.00 Ref		1.00 Ref	
Yes	0.35 (0.076–1.65)	0.354	0.26 (0.04–1.56)	0.139	0.35 (0.04–2.48)	0.308	0.14 (0.009–2.05)	0.151
**Risk perception**								
Will not happen/not likely	1.00 Ref		1.00 Ref		1.00 Ref		1.00 Ref	
Likely ti happen/definitely will happen	0.943 (0.49–1.81)	0.859	0.95 (0.46–1.98)	0.890	0.66 (0.30–1.44)	0.657	0.66 (0.25–1.73)	0.394
**UPPS-P (1-point increase)**								
Urgency	**1.07 (1.01–1.15)**	**0.032**	1.06 (0.99–1.16)	0.165	**1.17 (1.07–1.26)**	**<0.001**	1.08 (0.97–1.21)	0.185
Sensation seeking	0.99 (0.93–1.07)	0.884	1.006 (0.92–1.09)	0.891	0.98 (0.90–1.07)	0.652	0.98 (0.87–1.09)	0.652
Lack of perseverance	1.05 (0.92–1.18)	0.499	0.93 (0.79–1.09)	0.335	**1.24 (1.07–1.43)**	**0.004**	1.08 (0.88–1.32)	0.476
Lack of premeditation	1.04 (0.93–1.17)	0.508	1.07 (0.93–1.24)	0.335	**1.36 (1.17–1.59)**	**<0.001**	**1.31 (1.09–1.59)**	**0.005**
**PANAS (1-point increase)**								
Positive affect	0.86 (0.79–0.98)	**0.016**	**0.83 (0.73–0.96)**	**0.010**	0.95 (0.83–1.09)	0.466	1.04 (0.86–1.25)	0.725
Negative affect	1.02 (0.94–1.12)	0.615	0.95 (0.85–1.06)	0.386	1.09 (0.98–1.22)	0.125	0.99 (0.86–1.15)	0.993

*n* = 204; * Multinomial logistic regression with IESF as the dependent variable (category not susceptible as a reference) the other variables as independent variables. aOR: adjusted odds ratio. cOR: Crude odds ratio. 95% CI: 95% confidence interval. Ref.: Reference. ESIe-c: Expanded susceptibility index, electronic cigarettes. UPPS-P: Impulsive Behaviours Scale. PANAS: Positive and Negative Affective Scale.

## Data Availability

The authors confirm that all the data supporting the findings of this study are available within the article. The data that support the findings of this study are available from the corresponding author, upon reasonable request.

## Public Involvement Statement

There was no public involvement in any aspect of this research.

## Guidelines and Standards Statement

This manuscript was drafted in accordance with the STROBE statement guideline for observational studies.
